# Advancing a paradigm shift in evaluation of forensic evidence: The rise of forensic data science

**DOI:** 10.1016/j.fsisyn.2022.100270

**Published:** 2022-05-18

**Authors:** Geoffrey Stewart Morrison

**Affiliations:** a Forensic Data Science Laboratory, Aston University, Birmingham, UK; b Forensic Evaluation Ltd, Birmingham, UK

**Keywords:** Forensic science, Forensic data science, Likelihood ratio, Paradigm shift, Validation, HoL, House of Lords Science and Technology Select Committee, PCAST, President’s Council of Advisors on Science and Technology, EWG, Expert Working Group on Human Factors in Latent Print Analysis, FSR, Forensic Science Regulator for England & Wales, TRL, technology readiness level, UKRI, United Kingdom Research and Innovation

## Abstract

Widespread practice across the majority of branches of forensic science uses analytical methods based on human perception, and interpretive methods based on subjective judgement. These methods are non-transparent and are susceptible to cognitive bias, interpretation is often logically flawed, and forensic-evaluation systems are often not empirically validated. I describe a paradigm shift in which existing methods are replaced by methods based on relevant data, quantitative measurements, and statistical models; methods that are transparent and reproducible, are intrinsically resistant to cognitive bias, use the logically correct framework for interpretation of evidence (the likelihood-ratio framework), and are empirically validated under casework conditions.

## 1 Introduction

The present paper is a written version of a keynote presentation given at the European Academy of Forensic Science 2022 conference. It discusses an ongoing paradigm shift in evaluation of forensic evidence. It describes:

• the current state of affairs (*staus quo*)
• the new paradigm (*quo vadis?*)
• obstacles to the advancement of the paradigm shift (*impedimenta*)
• a strategy to advance the paradigm shift (*via progredi*)

## 2 A paradigm shift in evaluation of forensic evidence

### 2.1 Status quo

Curran [1]:

Is forensic science the last bastion of resistance against statistics?

UK House of Lords Science and Technology Select Committee (HoL) [2]:

In regard to pattern comparison methods, … “the comparison of fingerprints, toolmarks, footwear, tire marks and ballistics” [are] “spot-the-difference” techniques in which “there is little, if any, robust science involved in the analytical or comparative processes used and as a consequence there have been questions raised around the reproducibility, repeatability, accuracy and error rates of such analysis.” (§155)

In forensic science, the process of *evaluation of strength of evidence* consists of: *analysis*, i.e., extraction of information from items of interest (the evidence)^1^; and *interpretation*, i.e., drawing inferences with respect to the meaning of the information extracted by the analysis. Items of interest may be, for example:

• a fingermark of questioned source recovered from a crime scene and a fingerprint collected from a known individual
• a recording of a speaker of questioned identity on an intercepted telephone call and a recording of a police interview with a speaker of known identity
• a fired cartridge case recovered from a crime scene and cartridge cases fired in a forensic laboratory from a gun found in the possession of a suspected shooter

Forensic practitioners conduct evaluations in order to assist legal-decision makers to make decisions with respect to questions of legal concern such as:^2^

• Do the fingermark and fingerprint originate from the same finger?
• Is the speaker of questioned identity on the intercepted recording the same as the speaker of known identity?
• Was the cartridge case recovered from the crime scene fired from the suspect's gun?

Currently, across the majority of branches of forensic science, widespread practice is that analysis is conducted using *human perception*, and interpretation is conducted using *subjective judgement*. Even in branches of forensic science in which analysis is conducted using instrumental measurement, interpretation is commonly based on subjective judgement, e.g., by eyeballing graphical representations of the measured values. Human-perception-based analysis methods and subjective-judgement-based interpretation methods are non-transparent and are susceptible to cognitive bias.

Currently, across the majority of branches of forensic science, even branches of forensic science in which interpretation is conducted using statistical models, interpretation of evidence is often logically flawed, and forensic-evaluation systems (the end-to-end combination of analysis and interpretation methods) are often not empirically validated or not adequately empirically validated.^3^

### 2.2 Quo vadis?

#### 2.2.1 Introduction

Saks & Koehler [4]:

we envision a paradigm shift in the traditional forensic identification sciences in which untested assumptions and semi-informed guesswork are replaced by a sound scientific foundation and justifiable protocols. Although obstacles exist both inside and outside forensic science, the time is ripe for the traditional forensic sciences to replace antiquated assumptions of uniqueness and perfection with a more defensible empirical and probabilistic foundation. (p. 895)

US President's Council of Advisors on Science and Technology (PCAST [5]):

neither experience, nor judgment, nor good professional practice … can substitute for actual evidence of foundational validity and reliability. The frequency with which a particular pattern or set of features will be observed in different samples, which is an essential element in drawing conclusions, is not a matter of “judgment.” It is an empirical matter for which only empirical evidence is relevant. (p. 6)

Objective methods are, in general, preferable to subjective methods. Analyses that depend on human judgment (rather than a quantitative measure …) are obviously more susceptible to human error, bias, and performance variability across examiners. In contrast, objective, quantified methods tend to yield greater accuracy, repeatability and reliability, including reducing variation in results among examiners. Subjective methods can evolve into or be replaced by objective methods. (p. 47)

A *paradigm shift* in evaluation of forensic evidence is ongoing in which methods based on human perception and subjective judgement are replaced by methods based on *relevant data*, *quantitative measurements*, and *statistical models/machine-learning algorithms*; methods that:

• are *transparent and reproducible* (§2.2.2);
• are *intrinsically resistant to cognitive bias* (§2.2.3);
• use the *logically correct framework for interpretation of evidence* (the *likelihood-ratio framework*) (§2.2.4); and
• are *empirically validated under casework conditions* (§2.2.5).

I address each of these elements of the new paradigm in the following four subsections. They are, in turn, followed by a subsection which discusses the applicability to this paradigm shift of Kuhn's [6] description of a paradigm shift, and the implications thereof (§2.2.6).

#### 2.2.2 Transparency and reproducibility

Methods dependent on human perception and subjective judgement are intrinsically non-transparent and therefore not reproducible by others. Human introspection is often mistaken, hence a forensic practitioner's explanation of how they reached their conclusion may not reflect how they actually reached that conclusion (Edmond et al. [7]).

In contrast, methods based on data, quantitative measurement, and statistical models are transparent and reproducible: measurement (feature-extraction) and statistical-modelling/machine-learning methods can be described in detail, and data and software tools can potentially be shared with others.

#### 2.2.3 Cognitive bias

There has been a great deal of concern about cognitive bias in forensic science (National Research Council [8]; Expert Working Group on Human Factors in Latent Print Analysis, EWG [9]; Found [10]; Stoel et al. [11]; PCAST [5]; Edmond et al. [7]; Cooper & Meterko [12]; Expert Working Group on Human Factors in Handwriting Examination [13]; Spellman et al. [14]). Cognitive bias is subconscious bias, it cannot be controlled by strength of will.

Forensic practitioners are susceptible to cognitive bias when making perceptual observations: their degree of belief in the probability that a hypothesis is true can affect their analysis of the evidence. Since the output of the analysis is the input to the interpretation, bias in the former affects the latter. Forensic practitioners are susceptible to cognitive bias when they are making subjective judgements and are exposed to information that could influence their degree of belief in the probability that a hypothesis is true but that would not logically affect the probability of obtaining the evidence conditional on whether the hypothesis were true.

Some potentially biasing information is task-irrelevant and should be withheld from practitioners, but some potentially biasing information is task-relevant and practitioners employing human-perception and subjective-judgement methods will need to be exposed to it at some point in the evaluation process, e.g., practitioners who visually compare known-source fingerprints and questioned-source fingermarks must be exposed to both, but exposure to a higher-quality print may bias their analysis of ambiguous details in a lower-quality mark.

Systems in which the strength-of-evidence conclusion is directly the result of subjective judgement are particularly susceptible to cognitive bias.

Systems based on quantitative measurements and statistical models require subjective judgements on decisions such as whether the data used for training the system and the data used for validating the system are sufficiently representative of the relevant population for the case and sufficiently reflective of the conditions of the items of interest in the case so that the output of the system will be a meaningful answer to the question posed in the case and so that the results of the validation will provide a meaningful indication of the performance of the systems under the conditions of the case. These decisions, however, are made at the beginning of the process before the practitioner has analyzed the items of interest, hence the practitioner cannot know what effect these decisions will have on the strength-of-evidence conclusion. The remainder of the evaluation process is automated, hence not susceptible to cognitive bias.

#### 2.2.4 Likelihood-ratio framework

In current practice, interpretation of evidence is often logically flawed, e.g., it is based on the uniqueness or the individualization fallacy (Saks & Koehler [15]; Cole [16], [17]), and conclusions are often expressed categorically, e.g., “identification”, “inconclusive”, “exclusion” (i.e., posterior probability of 1 or 0 with respect to the same-source hypothesis, with “inconclusive” meaning no conclusion rather than an intermediate probability), or using some form of uncalibrated verbal posterior-probability scale, e.g., “identification”, “probable identification”, “inconclusive”, “probable exclusion”, “exclusion”. Jackson [18] and Kaye [19] review these and other commonly used but logically flawed conclusions.

In contrast, the likelihood-ratio framework is advocated as the logically correct framework for evaluation of evidence by the vast majority of experts in forensic inference and statistics (including: Aitken et al. [20]; Morrison et al. [21]; Morrison et al. [22]; with 31, 19 and 20 authors and supporters respectively), and by key organizations including:

• Association of Forensic Science Providers of the United Kingdom and of the Republic of Ireland [23].
• Royal Statistical Society (Aitken et al. [24])
• European Network of Forensic Science Institutes (Willis et al. [25])
• National Institute of Forensic Science of the Australia New Zealand Policing Advisory Agency (Ballantyne et al. [26])
• American Statistical Association (Kafadar et al. [27])
• Forensic Science Regulator for England & Wales (FSR) [28].

The likelihood-ratio framework requires assessment of:

• the probability of obtaining the evidence if one hypothesis were true versus
• the probability of obtaining the evidence if an alternative hypothesis were true

The two hypotheses must be mutually exclusive. One hypothesis should represent the position of the prosecution in the case, and the other the position of the defence, e.g., the fingermark of questioned origin was deposited by a finger of a particular known individual, versus the fingermark of questioned origin was deposited by a finger of some other individual selected at random from the relevant population. In this example:

• the numerator of the likelihood ratio quantifies the *similarity* between the mark and the print
• the denominator quantifies the *typicality* of the mark with respect to the relevant population

For continuously-valued data, likelihood ratios can be calculated as the ratio of two probability-density functions evaluated at the value of the evidence.^4^

#### 2.2.5 Empirical validation

Empirical validation under conditions reflecting those of the case to which a forensic-evaluation system is to be applied is the only way to know how well that system performs under the conditions of the case. The need for validation under casework conditions has been emphasized by FSR [31], and by PCAST [5]:

Without appropriate estimates of accuracy, an examiner’s statement that two samples are similar—or even indistinguishable—is scientifically meaningless: it has no probative value, and considerable potential for prejudicial impact. Nothing—not training, personal experience nor professional practices—can substitute for adequate empirical demonstration of accuracy. (p. 46)

Protocols for validating systems that output likelihood ratios have been developed, including metrics and graphics appropriate for representing the results of such validations (Meuwly [32]; Brümmer & du Preez [33]; Morrison [34]; Meuwly et al. [35]; Ramos et al. [36]; Morrison et al. [22]). Much of the latter work has been conducted in the context of forensic voice comparison, but the results are applicable across forensic science in general.

#### 2.2.6 A Kuhnian paradigm shift

The idea that evaluation of forensic evidence is undergoing a paradigm shift is not new. The most famous article heralding a paradigm shift is Saks & Koehler [4]. Allowing for differences in wording and level of detail and the passage of time, I believe that Saks & Koehler [4] and the present paper describe the same paradigm shift. In contrast to Saks & Koehler's [4] statement that they intended “paradigm shift” as a metaphor, however, I view the paradigm shift in evaluation of forensic evidence as a true *Kuhnian paradigm shift* (Kuhn [6]) in the sense that^5^

• it requires rejection of existing methods and the ways of thinking that underpin them,
• and rejection of the idea that progress can be made by incremental improvements to existing methods.
• Instead, it requires the wholesale adoption of an entire constellation of new methods and new ways of thinking.

That a paradigm shift requires the wholesale adoption of an entire constellation of new methods and new ways of thinking remains the case irrespective of whether one considers the shift to be from one paradigm to another or to be from a pre-paradigm to a paradigm period of science. As suggested in Saks & Koehler [4], a pre-paradigm period would seem to be a more accurate description of the *status quo*, with multiple traditions of evaluation of evidence used both within individual branches of forensic science and across different branches of forensic science, and hence there being no established widely-accepted overarching paradigm in use.

Some authors have used the term “paradigm shift” in relation to a single element or a subset of the elements of the paradigm shift as I have outlined it above, but I believe that all of these elements are required as part of the constellation. This may be viewed as a radical stance, and it faces resistance, but, over the last decade and a half, my colleagues and I have had substantial success in contributing to advancing this paradigm shift in forensic voice comparison.

### 2.3 Impedimenta

#### 2.3.1 Introduction

The paradigm shift in evaluation of forensic evidence is ongoing, but progress is slow or stalling for multiple reasons including the following:

• The new paradigm has only been adopted in a few branches of forensic science, and only by a minority of researchers and practitioners (§2.3.2).
• Only some elements of the new paradigm have been adopted as part of incremental change (§2.3.3).
• There is misunderstanding of the new paradigm and resistance to its adoption (§2.3.4).
• Research is often not informed by practice and has no impact on practice (§2.3.5).
• It is difficult to obtain funding for evidential-forensic-science research (§2.3.6).
• There are genuine practical impediments to implementing the new paradigm (§2.3.7).

I discuss each of these impediments in the following six subsections.

#### 2.3.2 The new paradigm has only been adopted in a few branches of forensic sciences, and only by a minority of researchers and practitioners

In the 1990s, the new paradigm was widely adopted for **forensic evaluation of DNA** (Foreman et al. [37]). Although the volume and importance of casework in this branch of forensic science makes it influential, single-source DNA profiles are invariant and discrete. They therefore have a very different structure from the continuously-valued data with within-source variability that results from analyses in most other branches of forensic science. The situation is more complex for low-template DNA and for DNA mixtures, but there is still a difference in data structure. Interpretation of DNA profiles is also dependent on well-developed theory of genetic inheritance, whereas interpretation in most branches of forensic science will have to be data driven (as is the case in machine learning in general, including in biometric applications). The potential for transfer of new-paradigm knowledge and methods from DNA to other branches of forensic science is therefore limited.

Since around 2000, a growing number of researchers and practitioners in **forensic voice comparison** have developed and adopted methods for calculation of likelihood ratios based on acoustic measurements and statistical models / machine-learning algorithms, and have developed and adopted methods for calibration and validation of likelihood-ratio systems under casework conditions. This has included adoption of state-of-the-art machine-learning approaches to automatic speaker recognition (Lee et al. [38]; Matějka et al. [39]; Villalba et al. [40]; Morrison et al. [41]; Morrison et al. [42]; Weber et al. [43]). At present, however, only a minority of practitioners have adopted the new paradigm. Survey results published in 2011, 2016, and 2019 (Gold & French [44]; Morrison et al. [45]; Gold & French [46]) suggest that, although the proportion of practitioners who have adopted human-supervised-automatic approaches and numeric likelihood ratios is growing, they are still a minority.^6^ In addition, inconsistent with the new paradigm, most respondents in the most recent survey who reported using a human-supervised-automatic approach also reported that they combined it with human-perception-based analysis and subjective-judgement-based interpretation.

Data in human-supervised-automatic approaches are continuously valued and have intrinsic within-source variability, a data structure shared with many other branches of forensic science. Compared to DNA, new-paradigm knowledge and methods from forensic voice comparison, including statistical models and calibration and validation methods, should therefore be easier to transfer to and adapt for use other branches of forensic science. For an example of such transfer and adaptation, see Basu et al. [47]. Forensic voice comparison is, however, a relatively niche branch of forensic science, which limits the extent to which developments in forensic voice comparison are noticed and adopted by researchers and practitioners in other branches of forensic science.

Curran [1] lamented that only 13% of laboratories surveyed used the likelihood-ratio framework for **glass evidence**, but this may be one of the highest rates of adoption after DNA. In many **other branches of forensic science** the rate of adoption of the likelihood-ratio framework by practitioners is near zero (Bali et al. [48]; Cole & Barno [49]).

#### 2.3.3 Only some elements of the new paradigm have been adopted as part of incremental change

Although in the short term adopting some elements of the new paradigm as part of incremental change may be a viewed as a step in the right direction, in the long term it may actually impede a paradigm shift.

Just because it is a transition between incommensurables, the transition between competing paradigms cannot be made a step at a time, … Like the gestalt switch, it must occur all at once (though not necessarily in an instant) or not at all. (Kuhn [6], p. 149)

Some practitioners **assign likelihood-ratio values based on subjective judgement**, and the values they assign are not subject to empirical calibration or empirical validation (see Risinger [50]; Morrison & Thompson [51]; Morrison et al. [52]). Some authors emphasize the logic of the likelihood-ratio framework and consider subjective assignment of likelihood ratio an acceptable end goal or consider it a step in the right direction, but such incremental steps potentially impede a paradigm shift which requires the abandonment of interpretation methods that are entirely dependent on subjective judgement.^7^ In addition, placing an emphasis on subjectivist concepts of probability is detrimental to attempts to encourage practitioners to adopt methods based on relevant data, quantitative measurements, and statistical models, and to adopt empirical validation under casework conditions (Morrison [54]).

The majority of **proposals to address cognitive bias** in forensic science (e.g., EWG [9]; Stoel et al. [11]; Thompson et al. [55]; FSR [56]) **assume the continued use of human-perception- and subjective-judgement-based methods**. Although this may be necessary in the short term, it potentially impedes a paradigm shift to quantitative-measurement- and statistical-model-based methods.

Some practitioners **make use of systems based on quantitative measurements and statistical models, but do not empirically calibrate or validate** their system using data that reflect the relevant population and the conditions for the case, and/or **rather than directly report the output of the system, they use it as input to a subjective-judgement process** that also considers other information including from human-perception-based analyses (see Morrison & Thompson [51]; Morrison [57], [58]). Such approaches are pernicious in that use of technology may give the false impression of scientific validity, and reaction against this may impede a paradigm shift that includes adoption of quantitative measurements and statistical models.

#### 2.3.4 There is misunderstanding of the new paradigm and resistance to its adoption

As with all Kuhnian paradigm shifts, there is misunderstanding of the new paradigm and resistance to its adoption.

**Some resitance is cultural**. The cultures of some branches of forensic science seem to be especially resistant to the adoption of statistical-model-based methods and of validation (see Mnookin et al. [59]; Curran [1]; Morrison [60]; Morrison & Stoel [61]; Swofford et al. [62]). Practitioners in multiple branches of forensic science often claim that training and experience provide sufficient warrant for their conclusions (see Mnookin et al. [59]; Risinger [50]; PCAST [5]; Morrison & Thompson [51]), and deny or obfuscate about the need for validation (see Cole [63]; Morrison [60]; PCAST [5]; Koehler [64]; Morrison et al. [52]), or propose lax validation protocols that do not require demonstration of performance under casework conditions (see Morrison et al. [65], [66]).

People in general tend to prefer methods which involve greater human input even when validation results indicate that data-driven methods lead to better results. Over time, however, people can come to accept data-driven methods (Swofford & Champod [67]).

There is a **belief that likelihood ratios are difficult to understand** (see Bali et al., 2020; Swofford et al. [62]; Swofford & Champod [68]). Commonly occurring misunderstandings have even been given names, e.g., the “prosecutor's fallacy” and the “defense attorney's fallacy” (Thompson & Schurmann [69]). There are many examples of legal rulings in which judges have misunderstood the meaning of a likelihood ratio (the England & Wales Court of Appeal 2010 ruling in *R v T* is an infamous example; see, e.g., Berger et al. [70]; Redmayne et al. [71]; Morrison [72]; Thompson [73]). Results of empirical research on lay understanding of expressions of strength of evidence are mixed (Eldridge [74]; Martire & Edmond [75]).

Despite legal rulings and recommendations concerning the need for validation, **courts often do not understand empirical validation and its necessity**, and accept testimony based on forensic-evaluation methods that have not been validated under conditions reflecting those of the case under consideration, or even that have not been empirically validated at all (see Bernstein [76]; Morrison [60], [57], [58]; Cooper [77]; Edmond [78]).

#### 2.3.5 Research is often not informed by practice and has no impact on practice

**Research that appears to be about forensic science may not actually be about solving real forensic-science problems**. For example, research may really be about a method in statistics or machine learning with an apparent forensic-science problem being used as an example of the application of that method.

Research in forensic science is sorely needed, but it should address primarily forensic science questions—not questions relating to the application of chemistry, biology, statistics, or psychology. (Margot [79], p. 801)

it is critical that researchers and funding bodies understand the importance of conducting research that is informed by practice and can be translated into practical applications. (Roux & Weyermann [80], p. 2)

Research that is divorced from forensic practice may lead to academic papers but nothing else. Even research that does address real forensic-science problems will fail to have impact unless it involves genuine collaboration in which researchers understand the demands of practice, and in which practitioners are willing to embrace research-informed change (Curran [1]).

#### 2.3.6 It is difficult to obtain funding for evidential-forensic-science research

**Few funding agencies have sustained funding targeted at forensic science**, and funding agencies seldom have panels of reviewers knowledgeable about evidential forensic science. Applications for funding for evidential-forensic-science research made to non-forensic-science-targeted calls are often rejected because reviewers do not understand the epistemology or value of forensic-science research. Applications are often rejected because their goals are to improve forensic science, which is an applied science, and **funding-agency criteria or reviewers often do not value applied science**.

The larger scientific community must now come to the aid of our forensic colleagues in advocating both for: (i) the research and financial support that is so clearly needed to advance the field and (ii) the requirement for empirical testing that is so clearly needed to advance the cause of justice …. Forensic scientists have long complained that their work is not always valued by their scientific colleagues because of its applied nature; it is time for the scientific community to move beyond that conceit. (Bell et al. [81])

At the other extreme, when there are calls specific to forensic science, they usually **focus exclusively or primarily on short-term goals related to law-enforcement investigative applications rather than on courtroom-evidential applications** (investigative and evidential applications have very different requirements), and they usually **focus on technology rather than on forensic inference**.

technology-oriented development … often overrul[es] the importance of appropriate scientific reasoning to solve actual problems (Roux et al. [82], p. 679)

Research calls requiring deliverables with a high technology readiness level (TRL) are not sources of funding for paradigm-shifting research.

In 2018, United Kingdom Research and Innovation (UKRI) informed the UK House of Lords Science and Technology Select Committee that UKRI had invested GBP 56 M over 10 years in forensic-science research (less than 0.1% of UKRI's total budget), but on closer inspection most of the funding counted to obtain that figure was not for research projects that actually focused on (or even made any contribution to) forensic science: only about GBP 17 M went to forensic-science focussed research, and GBP 15 M of that went to TRL research, only GBP 2 M to foundational research (HoL [2]; Morgan & Levin [83]). HoL [2] recommended that UKRI “urgently and substantially increase the amount of dedicated funding allocated to forensic science” (§187), but, more than 3 years after the publication of the HoL report, this has not (yet) happened.

#### 2.3.7 There are genuine practical impediments to implementing the new paradigm

Even if practitioners want to adopt the new paradigm, they will be unable to do so unless they are provided with the quantitative-measurement and statistical-modelling/machine-learning tools and the case-relevant data necessary to calculate likelihood ratios and validate systems under the conditions of the cases on which they work. Practitioners will also not be able to adopt the new paradigm unless they are provided with training on understanding the new paradigm in general and on how to implement it for the types of cases they work on.

### 2.4 Via progredi

Kuhn [6]:

The transfer of allegiance from paradigm to paradigm is a conversion experience that cannot be forced …. a generation is sometimes required to effect the change … Conversions will occur a few at a time until, after the last holdouts have died, the whole profession will again be practicing under a single, but now a different, paradigm. (pp. 150–151)

Kuhnian paradigm shifts are not rapid and individuals cannot be forced to embrace the new paradigm, but my aim is to facilitate and thereby advance the adoption of the new paradigm. My **strategy** is to work with researchers and practitioners who want to adopt the new paradigm, to work with them on addressing practical impediments to applying the new paradigm in casework, i.e.:

1. To provide researchers, practitioners, and lawyers with training leading to understanding of the new paradigm;
2. To collaborate with researchers and practitioners on building relevant databases and on developing and validating statistical models applicable in their particular branches of forensic science; and
3. To conduct research on how to present likelihood ratios and validation results so as to maximize understanding by laypeople, and thereby provide guidance to forensic practitioners on how to communicate forensic-evaluation results to legal-decision makers.

I invite others to consider whether this is a strategy that they would also be interested in adopting, either in part or in whole.

Element 2 of the strategy requires collaboration between researchers with expertise in forensic data science and researchers and practitioners with expertise in particular branches of forensic science. Academic publications are unlikely to convince practitioners to adopt the new paradigm, but other practitioners successfully applying the new paradigm are potentially convincing. In any branch of forensic science, the number of practitioners who initially want to adopt the new paradigm and who want to collaborate on this endeavour will almost certainly be a very small minority, but it will be more productive to work with a small minority on developing practical solutions than to try to convince the majority of practitioners without providing practical solutions. Once the practical solutions are being used by the small minority, use of the new paradigm has the potential to spread. Even then, I do not expect adoption of the new paradigm to be rapid, but I do expect higher rates of adoption among newer practitioners and trainees, leading to a generational shift.

## 3 Conclusion

A paradigm shift in evaluation of forensic evidence is ongoing. The shift is away from methods based on human perception and subjective judgement, to methods based on:

• relevant data,
• quantitative measurements,
• and statistical models.

New paradigm methods:

• are transparent and reproducible
• are intrinsically resistant to cognitive bias
• use the logically correct framework for interpretation of evidence (the likelihood-ratio framework)
• are empirically validated under casework conditions

This is a Kuhnian paradigm shift, which requires:

• rejection of existing methods and the ways of thinking that underpin them
• rejection of the idea that progress can be made by incremental improvements to existing methods
• the wholesale adoption of an entire constellation of new methods and new ways of thinking

Some branches of forensic science, such as forensic voice comparison, are more advanced in the paradigm shift than others. Knowledge gained in advancing the paradigm shift in forensic voice comparison can assist in advancing the paradigm shift in other branches of forensic science. Validation protocols, metrics, and graphics developed in the context of forensic voice comparison are immediately applicable in other branches of forensic science. Statistical models / machine-learning algorithms used in forensic voice comparison can even be transferred and adapted for use in other branches of forensic science.

My strategy for advancing the paradigm shift requires collaboration between researchers with expertise in forensic data science and researchers and practitioners with expertise in particular branches of forensic science. My strategy is to work with researchers and practitioners who want to adopt the new paradigm. My strategy is to work with them on addressing practical impediments to applying the new paradigm in casework, i.e.:

1. To provide researchers, practitioners, and lawyers with training leading to understanding of the new paradigm;
2. To collaborate with researchers and practitioners on building relevant databases and on developing and validating statistical models applicable in their particular branches of forensic science; and
3. To conduct research on how to present likelihood ratios and validation results so as to maximize understanding by laypeople, and thereby provide guidance to forensic practitioners on how to communicate forensic-evaluation results to legal-decision makers.

In any branch of forensic science, the number of practitioners who initially want to adopt the new paradigm and who want to collaborate on this endeavour will almost certainly be very small, but it will be more productive to work with a small minority on developing practical solutions than to try to convince the majority of practitioners without providing practical solutions. Once the practical solutions are being used by the small minority, use of the new paradigm has the potential to spread. Even then, I do not expect adoption of the new paradigm to be rapid, but I do expect higher rates of adoption among newer practitioners and trainees, leading to a generational shift.

I have been asked several times over the years whether I could suggest a name for the new paradigm other than “new”. Here, I propose that the new paradigm could be called “forensic data science”. My hope is that, after the paradigm shift is complete, it will simply be called “forensic science”.

## Disclaimer

All opinions expressed in the present paper are those of the author, and, unless explicitly stated otherwise, should not be construed as representing the policies or positions of any organizations with which the author is associated.

## Declaration of competing interest

The author declares that they have no known competing financial interests or personal relationships that could have appeared to influence the work reported in this paper.

## Footnotes

1 In the forensic-inference-and-statistics literature “evidence” is the term commonly used to refer to the items of interest (i.e., the input to the analysis) or to the information output by the analysis (i.e., the input to the interpretation). Usage is somewhat fluid, but, either way, this is evidence from the perspective of the forensic practitioner. From the perspective of the court, evidence is the oral testimony or written submission of the forensic practitioner. In wider forensic-science literature, the term “trace” is often used to refer to items of interest (Roux et al. [3]).

2 The present paper restricts its scope to the problem of source attribution.

3 Claims made in §2.1 are supported by details and references provided in §2.2 and in §2.3.

4 In the forensic inference and statistics literature “likelihood ratio” is commonly used as a cover term for both likelihood ratios based only on sample data and for Bayes' factors based on sample data and prior distributions for model parameters. The present discussion is intended to be neutral with respect to likelihoodist or Bayesian approaches. For simplicity, the present paper glosses over the differences between likelihoodist and Bayesian approaches. For readers particularly concerned with the differences between these two approaches, or with the nuances of one or the other of them, the present paper may be frustrating vague and such readers may feel that it conflates concepts that they considered to be importantly different. Addressing the concerns of such readers would, however, likely lead to greater confusion for the broader audience of forensic scientists and lawyers to whom the present paper is primarily addressed. Readers interested in the differences between likelihoodist and Bayesian approaches may wish to consult Ommen & Saunders [29,30].

5 The only aspects in which I think the current paradigm shift in evaluation of forensic evidence deviates from Kuhn's [6] description of paradigm shifts relate to forensic science being an applied science which is not isolated from societal pressures.

6 Percentages of respondents who reported that they used human-supervised-automatic approaches and numeric likelihood ratios were 20% and 9% respectively in the first survey, 33% and 23% respectively in the second, and 41% and 13% respectively in the third. Respondents in the first and third surveys included private practitioners and practitioners in government labs, and the results of these two surveys should be directly comparable. Respondents in the second survey were from a different population, law-enforcement agencies in INTERPOL member countries, which likely accounts for some of the difference between the results of this survey and those of the other two.

7 My stance on where in the forensic-evaluation process use of subjective judgement is acceptable is more restrictive than that of some leaders in the field of forensic inference and statistics, e.g., Evett et al. [53].

## References

[1] Curran J.M. (2013). Is forensic science the last bastion of resistance against statistics?. Sci. Justice.

[2] House of Lords Science and Technology Select Committee (2019). Forensic science and the criminal justice system: a blueprint for change. https://publications.parliament.uk/pa/ld201719/ldselect/ldsctech/333/333.pdf.

[3] Roux C., Bucht R., Crispino F., De Forest P., Lennard C., Margot P., Miranda M.D., NicDaéid N., Ribaux O., Ross A., Willis S. (2022). The Sydney declaration – revisiting the essence of forensic science through its fundamental principles. Forensic Sci. Int..

[4] Saks M.J., Koehler J.J. (2005). The coming paradigm shift in forensic identification science. Science.

[5] President’s Council of Advisors on Science and Technology (2016). Forensic science in criminal courts: ensuring scientific validity of feature-comparison methods. https://obamawhitehouse.archives.gov/administration/eop/ostp/pcast/docsreports/.

[6] Kuhn T.S. (1962).

[7] Edmond G., Towler A., Growns B., Ribeiro G., Found B., White D., Ballantyne K., Searston R.A., Thompson M.B., Tangen J.M., Kemp R.I., Martire K.A. (2017). Thinking forensics: cognitive science for forensic practitioners. Sci. Justice.

[8] National Research Council of the National Academies (2009).

[9] Expert Working Group on Human Factors in Latent Print Analysis (2012).

[10] Found B. (2015). Deciphering the human condition: the rise of cognitive forensics. Aust. J. Forensic Sci..

[11] Stoel R.D., Berger C.E.H., Kerkhoff W., Mattijssen E.J.A.T., Dror E.I., Strom K.J., Hickman M.J. (2015). Forensic Science and the Administration of Justice: Critical Issues and Directions.

[12] Cooper G.S., Meterko V. (2019). Cognitive bias research in forensic science: a systematic review. Forensic Sci. Int..

[13] Expert Working Group on Human Factors in Handwriting Examination (2020).

[14] Spellman B.A., Eldridge H., Bieber P. (2022). Challenges to reasoning in forensic science decisions. Forensic Sci. Int.: Synergy.

[15] Saks M.J., Koehler J.J. (2008). The individualization fallacy in forensic science. Vanderbilt Law Rev..

[16] Cole S.A. (2009). Forensics without uniqueness, conclusions without individualization: the new epistemology of forensic identification. Law Probab. Risk.

[17] Cole S.A. (2014). Individualization is dead, long live individualization! Reforms of reporting practices for fingerprint analysis in the United States. Law Probab. Risk.

[18] Jackson G., Fraser J., Williams R. (2009). Handbook of Forensic Science.

[19] Kaye D.H., Neumann C., Ranadive A., Kaye D.H. (2015). Communicating the Results of Forensic Science Examinations. Final Technical Report for NIST Award Number 70NANB12H014.

[20] Aitken C.G.G., Berger C.E.H., Buckleton J.S., Champod C., Curran J.M., Dawid A.P., Evett I.W., Gill P., González-Rodríguez J., Jackson G., Kloosterman A., Lovelock T., Lucy D., Margot P., McKenna L., Meuwly D., Neumann C., Nic Daéid N., Nordgaard A., Puch-Solis R., Rasmusson B., Redmayne M., Roberts P., Robertson B., Roux C., Sjerps M.J., Taroni F., Tjin-A-Tsoi T., Vignaux G.A., Willis S.M., Zadora G. (2011). Expressing evaluative opinions: a position statement. Sci. Justice.

[21] Morrison G.S., Kaye D.H., Balding D.J., Taylor D., Dawid P., Aitken C.G.G., Gittelson S., Zadora G., Robertson B., Willis S.M., Pope S., Neil M., Martire K.A., Hepler A., Gill R.D., Jamieson A., de Zoete J., Ostrum R.B., Caliebe A. (2017). A comment on the PCAST report: skip the “match”/“non-match” stage. Forensic Sci. Int..

[22] Morrison G.S., Enzinger E., Hughes V., Jessen M., Meuwly D., Neumann C., Planting S., Thompson W.C., van der Vloed D., Ypma R.J.F., Zhang C., Anonymous A., Anonymous B. (2021). Consensus on validation of forensic voice comparison. Sci. Justice.

[23] Association of Forensic Science Providers (2009). Standards for the formulation of evaluative forensic science expert opinion. Sci. Justice.

[24] Aitken C.G.G., Roberts P., Jackson G. (2010). https://rss.org.uk/news-publication/publications/law-guides/.

[25] Willis S.M., McKenna L., McDermott S., O'Donell G., Barrett A., Rasmusson A., Nordgaard A., Berger C.E.H., Sjerps M.J., Lucena-Molina J.J., Zadora G., Aitken C.G.G., Lunt L., Champod C., Biedermann A., Hicks T.N., Taroni F. (2015). ENFSI guideline for evaluative reporting in forensic science. http://enfsi.eu/wp-content/uploads/2016/09/m1_guideline.pdf.

[26] Ballantyne K., Bunford J., Found B., Neville D., Taylor D., Wevers G., Catoggio D. (2017). http://www.anzpaa.org.au/forensic-science/our-work/projects/evaluative-reporting.

[27] Kafadar K., Stern H., Cuellar M., Curran J.M., Lancaster M., Neumann C., Saunders C., Weir B., Zabell S. (2019). American statistical association position on statistical statements for forensic evidence. https://www.amstat.org/asa/files/pdfs/POL-ForensicScience.pdf.

[28] Forensic Science Regulator (2021). Codes of practice and conduct: development of evaluative opinions. https://www.gov.uk/government/publications/development-of-evaluative-opinions.

[29] Ommen D.M., Saunders C.P. (2021). A problem in forensic science highlighting the differences between the Bayes factor and likelihood ratio. Stat. Sci..

[30] Ommen D.M., Saunders C.P., Carriquiry A.L., Tanur J.M., Eddy W.F. (2022). Statistics in the Public Interest.

[31] Forensic Science Regulator (2020). Guidance: validation (FSR-G-201 issue 2). https://www.gov.uk/government/publications/forensic-science-providers-validation.

[32] Meuwly D. (2001). https://www.unil.ch/files/live/sites/esc/files/shared/These.Meuwly.pdf.

[33] Brümmer N., du Preez J. (2006). Application independent evaluation of speaker detection. Comput. Speech Lang.

[34] Morrison G.S. (2011). Measuring the validity and reliability of forensic likelihood-ratio systems. Sci. Justice.

[35] Meuwly D., Ramos D., Haraksim R. (2017). A guideline for the validation of likelihood ratio methods used for forensic evidence evaluation. Forensic Sci. Int..

[36] Ramos D., Meuwly D., Haraksim R., Berger C.E.H., Banks D., Kafadar K., Kaye D.H., Tackett M. (2020). Handbook of Forensic Statistics.

[37] Foreman L.A., Champod C., Evett I.W., Lambert J.A., Pope S. (2003). Interpreting DNA evidence: a review. Int. Stat. Rev..

[38] Lee K.A., Yamamoto H., Okabe K., Wang Q., Guo L., Koshinaka T., Zhang J., Shinoda K. (2020). NEC-TT System for mixed-bandwidth and multi-domain speaker recognition. Comput. Speech Lang.

[39] Matějka P., Plchot O., Glembek O., Burget L., Rohdin J., Zeinali H., Mošner L., Silnova A., Novotný O., Diez M., Černocký J.H. (2020). 13 years of speaker recognition research at BUT, with longitudinal analysis of NIST SRE. Comput. Speech Lang.

[40] Villalba J., Chen N., Snyder D., García-Romero D., McCree A., Sell G., Borgstrom J., García-Perera L.P., Richardson F., Dehak R., Torres-Carrasquillo P.A., Dehak N. (2020). State-of-the-art speaker recognition with neural network embeddings in NIST SRE18 and Speakers in the Wild evaluations. Comput. Speech Lang.

[41] Morrison G.S., Enzinger E., Ramos D., González-Rodríguez J., Lozano-Díez A., Banks D., Kafadar K., Kaye D.H., Tackett M. (2020). Handbook of Forensic Statistics.

[42] Morrison G.S., Weber P., Enzinger E., Labrador B., Lozano-Díez A., Ramos D., González-Rodríguez J., Houck M., Wilson L., Lewis S., Eldridge H., Reedy P., Lothridge K. (2022). Encyclopedia of Forensic Sciences.

[43] Weber P., Enzinger E., Labrador B., Lozano-Díez A., Ramos D., González-Rodríguez J., Morrison G.S. (2022). Validation of the alpha version of the E^3^ Forensic Speech Science System (E^3^FS^3^) core software tools. Forensic Sci. Int.: Synergy.

[44] Gold E., French J.P. (2011). International practices in forensic speaker comparison. Int. J. Speech Lang. Law.

[45] Morrison G.S., Sahito F.H., Jardine G., Djokic D., Clavet S., Berghs S., Goemans Dorny C. (2016). INTERPOL survey of the use of speaker identification by law enforcement agencies. Forensic Sci. Int..

[46] Gold E., French J.P. (2019). International practices in forensic speaker comparison: second survey. Int. J. Speech Lang. Law.

[47] Basu N., Bolton-King R.S., Morrison G.S. (2022). Forensic comparison of fired cartridge cases: feature-extraction methods for feature-based calculation of likelihood ratios. http://firearms.forensic-data-science.net/.

[48] Bali A.S., Edmond G., Ballantyne K.N., Kemp R.I., Martire K.A. (2020). Communicating forensic science opinion: an examination of expert reporting practices. Sci. Justice.

[49] Cole S.A., Barno M. (2020). Probabilistic reporting in criminal cases in the United States: a baseline study. Sci. Justice.

[50] Risinger D.M. (2013). Reservations about likelihood ratios (and some other aspects of forensic ‘Bayesianism’). Law Probab. Risk.

[51] Morrison G.S., Thompson W.C. (2017). Assessing the admissibility of a new generation of forensic voice comparison testimony. Columbia. Sci Technol. Law Rev..

[52] Morrison G.S., Ballantyne K., Geoghegan P.H. (2018). A response to Marquis et al (2017) what is the error margin of your signature analysis?. Forensic Sci. Int..

[53] Evett I.W., Berger C.E.H., Buckleton J.S., Champod C., Jackson G. (2017). Finding the way forward for forensic science in the US – a commentary on the PCAST report. Forensic Sci. Int..

[54] Morrison G.S. (2017). What should a forensic practitioner's likelihood ratio be? II. Sci. Justice.

[55] Thompson W.C., Black J., Jain A., Kadane J. (2017).

[56] Forensic Science Regulator (2020). Guidance: cognitive bias effects relevant to forensic science examinations (FSR-G-217 issue 2). https://www.gov.uk/government/publications/cognitive-bias-effects-relevant-to-forensic-science-examinations.

[57] Morrison G.S. (2018). Admissibility of forensic voice comparison testimony in England and Wales. Crim. Law Rev..

[58] Morrison G.S. (2018). The impact in forensic voice comparison of lack of calibration and of mismatched conditions between the known-speaker recording and the relevant-population sample recordings. Forensic Sci. Int..

[59] Mnookin J.L., Cole S.A., Dror I.E., Fisher B.A.J., Houck M.M., Inman K., Kaye D.H., Koehler J.J., Langenburg G., Risinger D.M., Rudin N., Siegel J., Stoney D.A. (2011). The need for a research culture in the forensic sciences. UCLA Law Rev..

[60] Morrison G.S. (2014). Distinguishing between forensic science and forensic pseudoscience: testing of validity and reliability, and approaches to forensic voice comparison. Sci. Justice.

[61] Morrison G.S., Stoel R.D. (2014). Forensic strength of evidence statements should preferably be likelihood ratios calculated using relevant data, quantitative measurements, and statistical models – a response to Lennard (2013) Fingerprint identification: how far have we come?. Aust. J. Forensic Sci..

[62] Swofford H., Cole S., King V. (2021). Mt. Everest – we are going to lose many: a survey of fingerprint examiners' attitudes towards probabilistic reporting. Law Probab. Risk.

[63] Cole S.A. (2006). Is fingerprint identification valid? Rhetorics of reliability in fingerprint proponents' discourse. Law Pol..

[64] Koehler J.J. (2017). Forensics or fauxrensics? Ascertaining accuracy in the forensic sciences. Ariz. State Law J..

[65] Morrison G.S., Neumann C., Geoghegan P.H. (2020). Vacuous standards – subversion of the OSAC standards-development process. Forensic Sci. Int.: Synergy.

[66] Morrison G.S., Neumann C., Geoghegan P.H., Edmond G., Grant T., Ostrum R.B., Roberts P., Saks M., Syndercombe Court D., Thompson W.C., Zabell S. (2021). Reply to Response to Vacuous standards – subversion of the OSAC standards-development process. Forensic Sci. Int.: Synergy.

[67] Swofford H., Champod C. (2021). Implementation of algorithms in pattern & impression evidence: a responsible and practical roadmap. Forensic Sci. Int.: Synergy.

[68] Swofford H., Champod C. (2022). Probabilistic reporting and algorithms in forensic science: stakeholder perspectives within the American criminal justice system. Forensic Sci. Int.: Synergy.

[69] Thompson W.C., Schumann E.L. (1987). Interpretation of statistical evidence in criminal trials: the prosecutor's fallacy and the defense attorney's fallacy. Law Hum. Behav..

[70] Berger C.E.H., Buckleton J., Champod C., Evett I.W., Jackson G. (2011). Evidence evaluation: a response to the Court of Appeal judgment in R v T. Sci. Justice.

[71] Redmayne M., Roberts P., Aitken C.G.G., Jackson G. (2011). Forensic science evidence in question. Crim. Law Rev..

[72] Morrison G.S. (2012). The likelihood-ratio framework and forensic evidence in court: a response to R v T. Int. J. Evid. Proof.

[73] Thompson W.C. (2012). Discussion paper: hard cases make bad law – reactions to R v T. Law Probab. Risk.

[74] Eldridge H. (2019). Juror comprehension of forensic expert testimony: a literature review and gap analysis. Forensic Sci. Int.: Synergy.

[75] Martire K.A., Edmond G., Banks D., Kafadar K., Kaye D.H., Tackett M. (2020). Handbook of Forensic Statistics.

[76] Bernstein D.E. (2013). The misbegotten judicial resistance to the Daubert revolution. Notre Dame Law Rev..

[77] Cooper S.L. (2016). Forensic science identification evidence: tensions between law and science. J. Philos. Sci. Law.

[78] Edmond G., Roberts P., Stockdale M. (2018). Forensic Science Evidence and Expert Witness Testimony.

[79] Margot P. (2011). Commentary on the need for a research culture in the forensic sciences. UCLA Law Rev..

[80] Roux C., Weyermann C. (2021). From research integrity to research relevance to advance forensic science. Forensic Sci. Res..

[81] Bell S., Sah S., Albright T.D., Gates S.J., Denton M.B., Casadevall A. (2018).

[82] Roux C., Willis S., Weyermann C. (2021). Shifting forensic science focus from means to purpose: a path forward for the discipline?. Sci. Justice.

[83] Morgan R.M., Levin E.A. (2019). A crisis for the future of forensic science: lessons from the UK of the importance of epistemology for funding research and development. Forensic Sci. Int.: Synergy.

